# Epidemiology and etiology of Traveler’s diarrhea in Bangkok, Thailand, a case-control study

**DOI:** 10.1186/s40794-019-0085-9

**Published:** 2019-06-07

**Authors:** Ladaporn Bodhidatta, Sinn Anuras, Siriporn Sornsakrin, Umaporn Suksawad, Oralak Serichantalergs, Apichai Srijan, Orntipa Sethabutr, Carl J. Mason

**Affiliations:** 1 Department of Enteric Diseases, Armed Forces Research Institute of Medical Sciences (AFRIMS), Bangkok, 10400 Thailand; 2 Bumrungrad International Hospital, Bangkok, 10110 Thailand; 3 Henry M. Jackson Foundation for the Advancement of Military Medicine, Bethesda, MD 20817 USA

**Keywords:** Traveler’s diarrhea, Enteric pathogens, Thailand, *Campylobacter*, *Vibrio*, *Shigella*, *Plesiomonas*, Norovirus

## Abstract

**Background:**

Traveler’s diarrhea (TD) is a common health problem among visitors from developed to developing countries. Although global and regional estimates of pathogen distribution are available, the etiology of diarrhea among non-military travelers to Thailand is largely unknown.

**Methods:**

A prospective TD case-control study was conducted among adult travelers from developed countries at a prominent hospital in Bangkok, Thailand during 2001–2003. Stool samples were collected from acute TD cases and non-diarrheal controls and analyzed for bacterial, viral, and protozoan pathogens by microbiology, ELISA or PCR methods. Calculation of adjusted odd ratios for risk factors was performed by logistic regression using STATA statistical software.

**Results:**

Stool samples were collected and analyzed from 389 TD cases and 400 non-diarrhea controls. At least one pathogen was detected in 227 (58%) cases and 124 (31%) controls. *Plesiomonas* (14%), *Vibrio* (14%), *Campylobacter* (14%), and norovirus (12%) were the most frequently isolated pathogens among cases and significantly associated with diarrhea at *p* = 0.006, *p* < 0.001, *p* < 0.001, *p* < 0.001, respectively. *Shigella* (3%) and ETEC (8%), detected in lower prevalence, also showed significant association with TD at *p* < 0.001 and *p* = 0.002, respectively. Travelers from East Asian countries had an increased risk of *Vibrio* infection (Crude odds ratio: 3.1, *p*-value = 0.001); travelers from the United States, Canada, and Europe had an increased risk of *Campylobacter* infection (Crude odds ratio: 2.6, *p*-value = 0.001); and travelers from Australia and New Zealand had an increased risk of *Salmonella* infection (Crude odds ratio: 3.2, *p*-value = 0.009).

**Conclusions:**

Etiology of TD in Thailand is mainly of bacterial origin. *Plesiomonas, Vibrio,* and norovirus are underappreciated diarrheagenic pathogens. In our study, the origin of the traveler plays an important role in diarrhea etiology. Understanding variations in TD severity and etiology among travelers from different regions warrants further study.

**Electronic supplementary material:**

The online version of this article (10.1186/s40794-019-0085-9) contains supplementary material, which is available to authorized users.

## Background

Traveler’s diarrhea (TD) is the most common health problem facing residents of developed countries who visit developing regions. Travelers most frequently develop TD within their first week(s) abroad [1–3]. Although TD in most cases resolves spontaneously within a few days without treatment and is unlikely to be fatal, it has a significant impact on quality of life and economics of healthcare service use, travel change expenses, loss of man-hours, and changes of vacation or business plans [4, 5]. As many as 40% of TD cases modify their activities, 23% seek medical treatment, and 1% require hospitalization [5–8].

A systematic review of global TD etiology reported Enterotoxigenic *Escherichia coli* (ETEC) and Enteroaggregative *E. coli* (EAEC) as the most common diarrhea-causing pathogens isolated in 30.4 and 19.0% of TD cases respectively [9]. However, significant regional variations in pathogen distributions have been reported. In Southeast Asia, *Campylobacter* (32.4%) and Enteropathogenic *E. coli* (EPEC) (18.0%) have been isolated most frequently, and multiple pathogen infections are apparently more common than in other regions [8, 9]. More detailed knowledge of regional pathogen distribution may enable more accurate predictions of vaccine-preventable disease and may also have implications for empiric treatment and pre-travel health advice.

The etiology of TD in adult travelers to Bangkok is largely unknown. Previous reports from Thailand on the etiology of TD largely focused on military populations whose unique risk profile, activities and travel history may differ from ordinary travelers and affect the TD epidemiology [5, 10–13]. Moreover, military deployments may not be ideal for surveillance of TD etiology in Thailand due to the limited numbers of subjects, short duration of travel, and restricted activities and locations. Studies of non-military TD in Thailand have focused on epidemiology or tested for a limited number of pathogens [14–19].

We conducted a prospective case-control study of patients presenting with TD to Bumrungrad International Hospital in Bangkok, Thailand during 2001–2003 in order to describe both TD etiology and epidemiology among adult residents of developed countries visiting Thailand.

## Methods

### Study population

Between January 2001 and January 2003, cases were recruited from adult residents of developed countries (Australia, Canada, Europe, Japan, South Korea, Taiwan, New Zealand and the United States) presenting with acute diarrhea of no more than 72 h duration to the Bumrungrad International Hospital in Bangkok, Thailand. Diarrhea was defined as three or more loose stools in the preceding 24 h with at least one additional symptom, such as nausea, vomiting, abdominal pain, fatigue, fecal urgency, or fever. Controls were recruited from adult residents of the countries mentioned above with no history of diarrhea in the preceding two weeks presenting to the Bumrungrad International Hospital for health screening or immunizations. Cases and controls were at least 20 years of age and were travelers or expatriates to Thailand.

Data regarding demographic, symptoms, antibiotic usage and travel history from both cases and controls were collected by study nurses. Each subject’s travel history was categorized by the highest risk destination visited during the seven days prior to diarrhea onset according to the country risk levels assigned by Greenwood et al. [20]. Subjects with missing or discrepant data were excluded from analysis.

Written informed consent was obtained from each participant prior to enrollment. This study was approved by the Institutional Review Board of the Walter Reed Army Institute of Research and the Ethical Review Committee for Research in Human Subjects, Ministry of Public Health, Thailand.

### Sample collection

Approximately 5 g of stool specimen were collected from each case and control. If a stool specimen was unobtainable, three rectal swabs were collected. Stool samples were transported to the nearby AFRIMS for processing within 4 h of collection. Three aliquots of one gram each were taken and stored at −70 °C for further testing.

### Microscopy and parasitology

Briefly, direct microscopic stool examination for red blood cells, white blood cells and parasites and a formalin-ether concentration technique for parasites was performed on each stool sample.

The microbiology techniques used in this study have been previously described elsewhere [21]. Briefly, stool was inoculated onto MacConkey (MC), Hektoen Enteric (HE), Thiosulfate Citrate Bile Salts Sucrose (TCBS), Modified Semisolid Rappaport Vassiliadis (MSRV) agar and enrichment media [Selenite F (SF), Alkaline Peptone Water (APW), Buffer Peptone Water (BPW), and Doyle]. Subsequently, subcultures from APW were plated on TCBS and subcultures from SF and BPW were plated onto MC, HE and MSRV. Subculture plates were incubated as previously described [21]. Culture for *Campylobacter* was performed both by inoculation of millipore filtered stool and by subculture of Doyle enrichment media after millipore filtration on Brucella agar (BA) with sheep blood and incubation under microaerobic conditions. *Campylobacter* isolates were identified at species level by using conventional phenotypic tests for *Campylobacter* [22]. *Shigella* and *Vibrio* isolates were subsequently serotyped using Denka-Seiken antisera (Denka-Seiken, Tokyo, Japan). *Salmonella* isolates were serogrouped using Serotest antisera (S&A Reagents Lab, Bangkok, Thailand).

Up to 5 lactose fermenting and 5 non-lactose fermenting *E. coli* as identified on MC agar were saved on Dorset egg yolk media slants. *E.coli* isolates were tested by hybridization with specific Digoxigenin-labeled polynucleotide probes: Heat-labile enterotoxin (LT) and heat-stable enterotoxin (ST) probes for ETEC; EIEC probe for Enteroinvasive *E.coli* (EIEC); Shiga-like toxin (SLT) I and SLT II probes for Shiga-like toxin producing *E.coli*; Effacing and Attaching *E.coli* (EAE), Entero adherence factor (EAF), and Bundle-forming protein A (BfpA) probes for EPEC [23–27]. ETEC colonization factor antigens (CFAs), coli surface (CS) and putative colonization factor antigens (PCF) antigens were detected by dot-blotting assays using monoclonal antibodies against CFA/I, CFA/III, CS1, CS2, CS3, CS4, CS5, CS6, CS7, CS17, PCFO159, PCFO166 as described previously [28, 29].

Detection of norovirus genogroup I and II was performed by a real-time reverse transcriptase PCR technique on available samples at AFRIMS [30]. All stool samples were tested for intestinal parasites by direct and concentrated microscopic examination. Samples with adequate quantity were further tested by ELISA. Common intestinal parasites, *Giardia lamblia* and *Cryptosporidium* were identified using commercial EIA kits (ProSpecT®, Remel, KS, USA). Samples positive on the combination *Giardia/Cryptosporidium* ELISA test were subsequently tested by *Giardia*-specific ELISA and *Cryptosporidium*-specific ELISA.

### Statistics

Data were analyzed using STATA 14 (STATA Corp LP, College Station, TX, USA). Isolation percentages were calculated for cases and controls and compared by chi-square or Fisher exact test with a significance level of *p* < 0.05. For continuous variables, means were compared by student t-test. Medians and categorical variable distributions were compared by Pearson chi-square statistic. A two-sided Fisher’s exact test was performed when zero cell counts excluded regression analysis. Crude odds ratios for demographic factors and symptoms were calculated using single variable logistic regression. Adjusted odds ratios were calculated by multivariate logistic regression including sex and age (year) and a categorical variable for length of time in Thailand.

## Results

### Study population

Of 417 cases and 417 controls enrolled, 389 cases and 400 controls with complete data and either a stool sample or rectal swabs were included for analysis. Compared to controls, cases were younger, predominantly female, and had been in Thailand for a shorter period of time (Table 1). Length of stay in Thailand ranged from 1 day to 40 years for both cases and controls, with 207 (53%) cases and 85 (21%) controls reporting a stay of less than 1 month. Cases were also more likely than controls to report travel to a moderate or high-risk location, however this difference was not significant after adjustment for sex, age, and time in Thailand (Crude odds ratio: 1.62, *p*-value = 0.003; Adjusted odds ratio: 1.03, *p*-value = 0.882).

**Table 1:** Subject characteristics comparing adult traveler’s diarrhea cases and non-diarrheal controls at Bumrungrad International Hospital in Bangkok, Thailand

	Case^1^	Control^1^	*p*-value^2^
Enrollees^3^	*N* = 389	*N* = 400	
Age (years)			
Mean ± SD	37.48 ± 13.71	41.6 ± 9.52	0.000
Gender			
Female	38% (32.95, 42.81)	25% (21.06, 29.81)	0.000
Nationality			
Asian	51% (45.81, 55.97)	51% (46.23, 56.25)	0.044
European / North American	42% (36.70, 46.72)	45% (40.30, 50.27)	
Oceania	7% (5.05, 10.53)	4% (1.93, 5.80)	
Time in Thailand			
Mean ± SD	540.43 ± 1410.75	1104.09 ± 1616.82	0.000
0–29 days	53% (48.12, 58.26)	21% (17.34, 25.59)	0.000
30–89 days	6% (4.20, 9.34)	3% (1.38, 4.87)	
90–364 days	13% (9.47, 16.31)	11% (7.67, 13.93)	
365–729 days	6% (4.20, 9.34)	17% (13.68, 21.32)	
730+ days	22% (17.37, 25.75)	48% (43.26, 53.27)	
Season at enrollment			
Hot: Feb – May	44% (38.96, 49.05)	38% (33.46, 43.21)	0.219
Rainy: Jun – Oct	40% (34.95, 44.90)	46% (40.54, 50.52)	
Cool: Nov – Jan	16% (12.68, 20.24)	16% (12.77, 20.24)	

^1^Percentage with 95% Confidence Intervals from binomial exact test

^2^Pearson Chi Square test for categorical variables, student t-test for continuous variables

^3^Additional 28 cases and 17 controls excluded for missing or discrepant data

One hundred and seventy-nine (46%) cases reported taking at least one medication for their diarrhea before stool sample collection. One hundred and forty-two (37%) of the cases received an antibiotic, of which majority of them were a quinolone. Seven cases reported taking an anti-parasitic, and six reported taking an anti-motility drug. In contrast to cases, only one control reported taking an antibiotic, of unknown type, in the preceding two weeks.

One hundred and eighty-five (48%) cases reported diarrhea onset of ≤24 h prior to hospital visits. Cases reported a range of bowel movements per 24 h of 3 to 30, with a median of 8.327. In regard to symptoms among TD cases: abdominal pain, nausea, fever, vomiting were reported in 84, 69, 58 and 53%, respectively. In TD cases, 294 (76%) reported watery stool, and 80 (21%) and 16 (4%) reported stool with mucus or blood, respectively.

### Pathogen identification

At least one pathogen was detected in 227 (58%) cases and 124 (31%) controls (*p* < 0.001). Detection of multiple pathogens was more common in cases (19%) than in controls (5%). Percent detection was significantly higher in cases than controls for *Campylobacter* (*p* < 0.001), *Shigella* (*p* < 0.001), *Vibrio* (*p* < 0.001), *Plesiomonas* (*p* = 0.006), ETEC (*p* = 0.002), and norovirus (*p* < 0.001) (Table 2). The most commonly isolated pathogens in cases were *Plesiomonas* (14%), *Campylobacter* (14%), *Vibrio* (14%) and norovirus (12%). *Salmonella* (13%) and *Plesiomonas* (8%) were the most frequently isolated pathogens in controls.

**Table 2:** Detection of enteric organisms in adult traveler’s diarrhea cases and non-diarrheal controls in Bangkok, Thailand

	Case^1^	Control^1^	*p*-value^2^
	*N* = 389	*N* = 400	
Pathogen isolated	227 (58%)	124 (31%)	0.000
Multiple pathogens isolated	74 (19%)	21 (5%)	0.000
Number of pathogens isolated			
0	162 (42%)	276 (69%)	0.000
1	153 (39%)	103 (26%)	
2	50 (13%)	19 (5%)	
3	18 (5%)	2 (0.5%)	
4	6 (1%)	0 (0%)	
Bacteria	198 (51%)	118 (30%)	0.000
*Plesiomonas*	55 (14%)	32 (8%)	0.006
*Campylobacter*	53 (14%)	8 (2%)	0.000
*Vibrio*	53 (14%)	1 (0.3%)	0.000
*Salmonella*	46 (12%)	50 (13%)	0.772
*Aeromonas*	18 (5%)	14 (4%)	0.422
*Shigella*	12 (3%)	0 (0%)	0.000
*Arcobacter*	1 (0.3%)	0 (0%)	0.493^3^
ETEC	26/325 (8%)	10/361 (3%)	0.002
EPEC	16/333 (5%)	19/373 (5%)	0.860
EIEC	3/333 (1%)	0/373 (0%)	0.104^3^
STEC	1/333 (0.3%)	4/373 (1%)	0.377^3^
Virus	32 (8%)	1 (0.3%)	0.000
Norovirus	32/259 (12%)	1/292 (0.3%)	0.000
Parasite	13 (3%)	8 (2%)	0.242
*Giardia*	10/363 (3%)	8/399 (2%)	0.496
*Cryptosporidium*	3/363 (0.7%)	0/399 (0%)	0.108^3^

^1^Percentage calculated as number of positive stool samples divided by number of samples tested for that pathogen. Some stool samples had more than one isolate

^2^Pearson Chi Square test

^3^Fisher’s exact test

Of the 56 isolates of *Vibrio spp.* from 54 stool samples, 49 (88%) isolates were *V. parahaemolyticus* (48 cases and 1 control), 5 (9%) *V. cholerae* Non O1/O139 (all cases), 1(2%) *Vibrio* group F (case), and 1(2%) *V. cholerae* O139 (case). Of the 63 *Campylobacter* isolates from 61 stool samples, 17% were *C. coli*, 78% were *C. jejuni*, 1 was *C. upsaliensis*, and 2 isolates were unknown type species. Of the 12 *Shigella* isolates, 9 (75%) were *S. sonnei* and others were *S. boydii* (2 cases) and *S. flexneri* 2a (1 case).

Enterotoxin genes for the 37 ETEC isolates from 36 cases or controls were also classified, with 7 positive for LT, 6 for STIa, 16 for STIb, 2 for LTSTIa, and 6 for LTSTIb. Thirty-six of the 37 ETEC isolates were tested for CFAs. Eighteen (50%) were positive with CS6 (19%), CS2,3 (14%), PCFO159 (5%), and CS1,3, CS17, PCFO166 and CFA/I (3% of each). The distribution of toxin genes was not significantly different between ETEC positive cases and controls.

A total of 20 samples were positive on the combination *Giardia/Cryptosporidium* ELISA test. Seventeen samples subsequently tested positive for *Giardia*-specific ELISA and 3 others for *Cryptosporidium*-specific ELISA. One additional sample of a case was positive for *Giardia* by direct and concentrated microscopy. These 21 samples were considered positive for presence of protozoa. Of the 32 cases with norovirus detection by PCR, 29 (91%) belonged to GII genogroup and 3 (9%) belonged to GI genogroup. One control was positive for GII norovirus.

### Regression analysis

Pathogen detection was more common in cases than in controls (Crude odds ratio: 3.09, *p*-value < 0.001, Adjusted odds ratio: 2.86, *p*-value < 0.001). Among cases, pathogen detection was more likely among those with moderate white blood cells (Crude odds ratio: 1.94, *p*-value = 0.053, Adjusted odds ratio: 1.81, *p*-value = 0.095), or many white blood cells (Crude odds ratio: 3.48, *p*-value < 0.001, Adjusted odds ratio: 3.49, *p*-value < 0.001) or many red blood cells (Crude odds ratio: 4.80, *p*-value < 0.001, Adjusted odds ratio: 5.06, *p*-value < 0.001) observed in the stool microscopic examination as compared to those with no blood cells in the stool. Prior antibiotic use decreased the likelihood of pathogen isolation (Crude odds ratio: 0.71, *p*-value = 0.110, Adjusted odds ratio: 0.68, *p*-value = 0.077). Compared to younger cases, those 25 and older were less likely to have a pathogen isolated (Crude odds ratio: 0.53, *p*-value = 0.030; Adjusted odds ratio: 0.55, *p*-value = 0.049). However, when prior antibiotic use was controlled for, the associations between pathogen isolation and age was no longer significant. Associations between pathogen isolation and sex, nationality, time in Thailand, season, diarrhea duration, mean bowel movement, and other symptoms were not statistically significant (Additional file 1: Table S1).

Isolation of bacteria was positively associated with the presence of many white blood cells (Crude odds ratio: 3.49, *p*-value < 0.001, Adjusted odds ratio: 3.48, *p*-value < 0.001) and many red blood cells (Crude odds ratio: 4.38, *p*-value < 0.001, Adjusted odds ratio: 4.72, *p*-value < 0.001). Reports of abdominal pain were also associated with an increased percentage of bacteria isolation (Crude odds ratio: 1.95, *p*-value = 0.019, Adjusted odds ratio: 1.93, *p*-value = 0.023). Use of antibiotics was negatively associated with the isolation of bacteria (Crude odds ratio: 0.72, *p*-value = 0.126, Adjusted odds ratio: 0.70, *p*-value = 0.091) but was not significant (Additional file 1: Table S2).

Norovirus detection was positively associated with reports of vomiting (Crude odds ratio: 4.01, *p*-value = 0.002, Adjusted odds ratio: 4.10, *p*-value = 0.002). Those presenting with diarrhea 3 days after onset were less likely than those with a diarrhea duration one day or less to have a virus detected (Crude odds ratio: 0.30, *p*-value = 0.012, Adjusted odds ratio: 0.27, *p*-value = 0.008) (Additional file 1: Table S3).

Protozoa detection was associated with travel to high-risk destinations [compare to low-risk/Bangkok destination] (Crude odds ratio: 11.81, *p*-value = 0.001, Adjusted odds ratio: 11.06, *p*-value = 0.005) and negatively associated with reports of abdominal pain (Crude odds ratio 0.147, *p*-value = 0.001, Adjusted odds ratio: 0.14, *p*-value = 0.001). Those presenting with diarrhea of 3 days duration were more likely to have protozoa detected than those presenting within the first day (Crude odds ratio: 4.59, *p*-value = 0.024, Adjusted odds ratio: 5.00, *p*-value = 0.019). After adjustment, protozoa detection was more likely in the rainy season (Crude odds ratio: 9.25, *p*-value = 0.037, Adjusted odds ratio: 10.06, *p*-value = 0.032) and the cool season (Crude odds ratio 11.53, *p*-value = 0.030, Adjusted odds ratio: 14.20, *p*-value = 0.020) as compared to the hot season (Additional file 1: Table S4). Gender, time in Thailand, antimotility drug use, antiparasitic drug use, and other diarrhea symptoms were not significantly associated with bacteria, viral or protozoan detection (Additional file 1).

Of the six pathogens with significantly higher detection percentages in cases, *Campylobacter*, ETEC and norovirus were associated with specific symptoms. As compared to other cases, those with *Campylobacter* isolated were more likely to report loose stool (22 of 53, Crude odds ratio 2.56, *p*-value = 0.002) or bloody stool (5 of 53, Crude odds ratio: 3.08, Fisher’s exact *p*-value = 0.052). Cases with ETEC isolated were less likely to report fever (9 of 26, Crude odds ratio: 0.36, *p*-value = 0.017). When norovirus was the only pathogen detected that compared with no any pathogen identified, cases were more likely to report the symptom of vomiting (18 of 19, crude odds ratio: 19.14, *p*-value = 0.005) and fatigue (14 of 19, crude odds ratio: 3.05, *p*-value = 0.040).

Moderate or many white blood cells (as compare with no blood cells) on microscopic stool examination were more often found in cases with *Campylobacter* (Crude odds ratio: 6.32, *p*-value < 0.001) and *Shigella* (Crude odds ratio: 9.56, *p*-value = 0.032) and found less often in cases where ETEC was the only pathogen isolated (Crude odds ratio: 0.24, *p*-value = 0.073) (Table 3). Moderate or many red blood cells were found more often in cases with *Campylobacter* (Crude odds ratio: 2.42, *p*-value = 0.012), *Shigella* (Crude odds ratio: 19.50, *p*-value = 0.005), *Vibrio* (Crude odds ratio: 2.69, *p*-value = 0.008), and *Plesiomonas* (Crude odds ratio: 2.29, *p*-value = 0.017). Moderate or many red blood cells were less often found in cases with ETEC (Crude odds ratio: 0.10, *p* = 0.023) (Table 3).

**Table 3:** Stool microscopic examination for WBCs and RBCs for 6 pathogens with significantly higher detection percentages in cases than in controls

	Moderate or many WBCs compare to negative blood cell		Moderate or many RBCs compare to negative blood cell	
	N (%)	Adjusted OR^a^ (95%CI)	N (%)	Adjusted OR^a^ (95%CI)
*Campylobacter*	34/51 (67%)	6.39 (2.52, 16.19)	19/51 (37%)	2.38 (1.15, 4.91)
*Shigella*	10/12 (83%)	9.76 (1.21, 78.96)	8/12 (67%)	22.30 (2.69, 184.58)
*Vibrio*	26/47 (55%)	2.47 (1.15, 5.34)	17/47 (36%)	2.73 (1.28, 5.82)
*Plesiomonas*	25/51 (49%)	1.76 (0.85, 3.66)	19/51 (37%)	2.30 (1.14, 4.62)
ETEC	7/25 (28%)	0.56 (0.20, 1.58)	1/25 (4%)	0.10 (0.01, 0.76)
Norovirus	13/32 (41%)	1.27 (0.51, 3.13)	9/32 (28%)	1.10 (0.45, 2.68)

^a^Adjusted by age (years), sex, and a categorical variable for time in Thailand

Compared to Europeans, North Americans, Australians and New Zealanders, Asian nationals were younger (mean age 35.31 years for case vs. 39.03 years for control, student t-test < 0.001), and they had been in Thailand for a shorter period of time (median stay 10 days for Asian case vs. 30 days for non-Asian case, median test: *p*-value = 0.001). Asian cases were more likely to have *Vibrio* isolated (Crude odds ratio: 3.10, *p*-value = 0.001, Adjusted odds ratio: 3.36, *p*-value < 0.001). *Campylobacter* was isolated more frequently in North Americans and Europeans (Crude odds ratio: 2.65, *p*-value = 0.001, Adjusted odds ratio: 2.79, *p*-value = 0.001). *Salmonella* was more frequently isolated in Australians and New Zealanders (Crude odds ratio: 3.23, *p*-value = 0.009, Adjusted odds ratio: 2.99, *p*-value = 0.018), and *Campylobacter* was isolated less frequently (none positive) (Fisher’s exact test *p*-value = 0.021) (Additional file 1: Table S5).

## Discussion

Our findings suggest that *Plesiomonas*, *Vibrio, Campylobacter*, and norovirus are important pathogens causing acute diarrhea among travelers and expatriates to Bangkok. Isolation percentages for *Shigella*, *Salmonella*, *Aeromonas*, ETEC, EIEC, *Giardia*, *Cryptosporidium,* and rotavirus were within three percentage points of the Southeast Asian regional estimates from Shah et al. [9]. Our data also supports Riddle’s finding that TD cases in Southeast Asia have a high risk of co-infection [8]. Unlike in Nepal, protozoa appear to cause little traveler’s diarrhea in travelers to Thailand [31], and cases with protozoa detected in this study were mainly associated with travel to a high-risk destination prior to visiting Thailand. However, it is important to note that 46% of TD cases in this study took medications including antibiotics for their diarrhea treatment prior to enrollment. This may have had potential effect on detection of bacterial organisms in stool samples by culture method.

The percent of *Campylobacter* isolation in our study was lower than an estimate of 32.4% in the review by Shah [9], possibly due to the large proportion of American military studies in the review. American soldiers may be at increased risk of *Campylobacter* infection, as was seen in the North American and European travelers in this study. Our study also found a four-fold higher percent detection for norovirus (12% vs. 0.3%). However, the odds of experiencing diarrhea given viral pathogen detection was much greater than for those with bacteria or protozoa isolation in this study, suggesting that norovirus is highly infective in addition to being common among travelers to Bangkok. As sapovirus, adenovirus, astrovirus were not tested in this study; the importance of viral pathogens in TD in Thailand may still be underappreciated.

Our study also suggests that composition of diarrhea etiologic agents varies by the traveler’s nationality. The odds of isolating *Vibrio* was significantly higher among Asian cases, while the odds of isolating *Campylobacter* was significantly higher among Europeans and North Americans and the odds of isolating *Salmonella* was significantly higher among Australians and New Zealanders.

Australians and New Zealanders may also suffer more severely from TD in Thailand than North Americans, Europeans and Asians [14, 19]. A previous survey study in Thailand by Chongsuvivatwong et al. of 22,401 travelers departing from Phuket or Chiang Mai including 2268 Australians and New Zealanders found Australians and New Zealanders to be at 2 to 3 times the risk of TD compared to Europeans and North Americans and to report experiencing more severe symptoms [14]. A subsequent survey by Kittatrakul et al. of 7963 travelers departing from Bangkok including 696 Australians and New Zealanders also found Australians and New Zealanders to be at greater risk of TD [19]. In our study, travelers from Australia and New Zealand were more likely to report fever, vomiting, and nausea. They were also less likely to have been in Thailand for one year or longer, thereby shortening their opportunity to develop natural immunity. However, in contrast to expectations for a naïve population, the percent isolation of *Campylobacter* among Australians and New Zealanders was significantly lower than Asians, North Americans and Europeans. An investigation of pathogen distribution by traveler nationality in other destinations and an exploration of risk factors, including dietary preferences and accommodation, may further elucidate the patterns of TD observed in this study.

Recruitment of cases and controls from developed countries in a prominent hospital in Bangkok may have biased our subjects towards well-off travelers with moderate to severe diarrhea. The pathogen distribution may not be representative of TD among all international visitors to Thailand. Other weaknesses of the study included the demographic differences between cases and controls, especially with regards to time in Thailand, the inability to determine the causal agent of co-infected patients, the inability to precisely determine the location of pathogen acquisition, the limited pathogen testing of some samples, samples were not tested for EAEC or the aforementioned viruses. Furthermore, the data were collected over 15 years ago and epidemiology of TD in Thailand may have changed.

Despite these limitations, our findings suggest that facility-based surveillance of TD in Thailand is feasible. Logistically, military deployments present several challenges to ongoing surveillance, and our studies suggest that their pathogen distribution may vary considerably from that of Thailand’s traveler population [5, 8–13]. Other TD studies have largely focused on the pathogen distribution and risk factors associated with specific travel destinations [7, 9]; however results from our study suggest that the origins of the traveler may play an important role in diarrhea etiology. More detailed characterization of the risk factors and pathogen distribution variations among travelers from different regions as well as a larger sample size and data collected from other locations will be required for better understanding of this observation. Understanding TD etiology and epidemiology will improve pre-travel health advice, empiric treatment and estimates of vaccine-preventable disease in this population. However, facility-based studies do not capture milder episodes of diarrhea or episodes that resolve by self-treatment, therefore a full estimate of TD disease burden will require complementary population-based research.

## Conclusions

Etiology of TD in Thailand is mainly caused by bacterial origin. *Plesiomonas, Vibrio*, and norovirus underappreciated as diarrheagenic pathogens. Although TD studies often focus on the pathogen distribution or risk factors associated with specific travel destinations, our study also confirms that the origin of the traveler also plays an important role in diarrhea symptoms and etiology. More detailed characterization of the risk factors and pathogen distribution variations among travelers from different regions warrants further study.

## Additional file

Additional file 1: Regression analyses for pathogen detection, bacterial isolation, norovirus detection, protozoa detection, and traveler origin. Supplementary tables of regression analyses results. (DOCX 44 kb)

## Abbreviations

CFAs: Colonization Factor Antigens
EAEC: Enteroaggregative *E. coli*
EIEC: Enteroinvasive *E.coli*
ELISA: Enzyme-linked immunosorbent assay
EPEC: Enteropathogenic *E.coli*
ETEC: Enterotoxigenic *E.coli*
LT: heat-labile enterotoxin
SLT: Shiga-like toxin
ST: heat-stable enterotoxin
STEC: Shiga toxin-producing *E. coli*
TD: Traveler’s diarrhea

## Authors’ information

Material has been reviewed by the Walter Reed Army Institute of Research. There is no objection to its publication. The opinions or assertions contained herein are the private views of the author, and are not to be construed as official, or as reflecting true views of the Department of the Army or the Department of Defense. The investigators have adhered to the policies for protection of human subjects as prescribed in AR 70–25.
