# Supplementary material for: Epidemiology and etiology of Traveler’s diarrhea in Bangkok, Thailand, a case-control study
Source: Trop Dis Travel Med Vaccines. 2019 Jun 7;5:9. doi: 10.1186/s40794-019-0085-9 (PMC6555966; doi:10.1186/s40794-019-0085-9)
Supplement: Supplementary file 1 — Regression analyses for pathogen detection, bacterial isolation, norovirus detection, protozoa detection, and traveler origin. Supplementary tables of regression analyses results. (DOCX 44 kb) [file 40794_2019_85_MOESM1_ESM.docx]

Additional File 1. Regression analyses for pathogen detection, bacterial isolation, norovirus detection, protozoa detection, and traveler origin.

Table S1. Crude and adjusted odds ratios for factors associated with pathogen detection in travelers’ diarrhea cases

|  | Case (n=389) | Control (n=400) |  | |
| --- | --- | --- | --- | --- |
|  | Number (%) | Number (%) | Crude OR (95% CI) | Adjusted OR (95% CI)* |
| **Pathogen detected** | **226 (58%)** | **124 (31%)** | **3.086 (2.304, 4.134)** | **2.864 (2.076, 3.952)** |

|  | Diarrhea Case with Pathogen Detection (n=226) | Diarrhea Cases without Pathogen Detection (n=163) |  | |
| --- | --- | --- | --- | --- |
|  | Number (%) | Number (%) | Crude OR (95% CI) | Adjusted OR (95% CI)* |
| Sex (female) | 89 (39%) | 58 (36%) | 1.176 (0.775, 1.785) | 1.110 (0.724, 1.701)** |
| Traveler ≥ 25 years old | 179 (79%) | 143 (88%) | 0.533 (0.302, 0.940) | 0.555 (0.309, 0.996)^†^ |
| Nationality  Asian  European/North American  Oceania | 120 (53%)  93 (41%)  13 (6%) | 78 (48%)  69 (42%)  16 (10%) | 1  0.876 (0.574, 1.336)  0.528 (0.241, 1.158) | 1  0.940 (0.607, 1.454)  0.530 (0.239, 1.174) |
| Time in Thailand  0 - 29 days  30 - 89 days  90 - 364 days  365 - 729 days  730+ days | 124 (55%)  16 (7%)  26 (12%)  13 (6%)  47 (21%) | 83 (51%)  9 (6%)  23 (14%)  12 (7%)  36 (22%) | 1  1.190 (0.502, 2.820)  0.757 (0.405, 1.415)  0.725 (0.315, 1.667)  0.874 (0.522, 1.463) | 1  1.153 (0.485, 2.740)^††^  0.769 (0.409, 1.447)^††^  0.709 (0.308, 1.634)^††^  0.927 (0.550, 1.563)^††^ |
| Season  Hot  Rainy  Cool | 94 (42%)  99 (44%)  33 (15%) | 77 (47%)  56 (34%)  30 (18%) | 1  1.448 (0.928, 2.260)  0.901 (0.505, 1.608) | 1  1.435 (0.916, 2.247)  0.927 (0.516, 1.664) |
| Diarrhea duration  ≤ 1 day  2 days  3 days | 114 (50%)  46 (20%)  66 (29%) | 71 (44%)  30 (18%)  62 (38%) | 1  0.955 (0.553, 1.650)  0.663 (0.420, 1.046) | 1  0.964 (0.549, 1.692)  0.658 (0.414, 1.046) |
| Bowel movements/day  (mean (95%CI)) | 8.46 (7.92, 9.01) | 8.18 (7.56, 8.80) | 1.018 (0.968, 1.070) | 1.021 (0.970, 1.075) |
| Stool characteristics  Watery  Loose  Mucous  Bloody | 170 (75%)  56 (25%)  48 (21%)  10 (4%) | 124 (76%)  39 (24%)  32 (20%)  6 (4%) | 0.955 (0.597, 1.527)  1.047 (0.655, 1.675)  1.104 (0.669, 1.822)  1.211 (0.431, 3.403) | 0.978 (0.608, 1.573)  1.023 (0.636, 1.645)  1.102 (0.665, 1.827)  1.190 (0.417, 3.393) |
| Symptoms  Fever  Abdominal pain  Nausea  Vomiting  Fatigue | 132 (58%)  192 (85%)  159 (70%)  128 (57%)  118 (52%) | 94 (58%)  135 (83%)  111 (68%)  79 (48%)  78 (48%) | 1.031 (0.685, 1.550)  1.171 (0.678, 2.023)  1.112 (0.719, 1.719)  1.389 (0.927, 2.081)  1.191 (0.796, 1.782) | 1.052 (0.692, 1.599)  1.136 (0.654, 1.972)  1.073 (0.686, 1.677)  1.359 (0.899, 2.055)  1.188 (0.791, 1.784) |
| White blood cell detection  Negative  Few  Moderate  **Many** | 62/213 (29%)  46/213 (22%)  31/213 (15%)  **74/213 (35%)** | 70/150 (47%)  38/150 (25%)  18/150 (12%)  **24/150 (16%)** | 1  1.367 (0.789, 2.367)  1.944 (0.991, 3.815)  **3.481 (1.962, 6.177)** | 1  1.332 (0.763, 2.324)  1.805 (0.903, 3.609)  **3.493 (1.948, 6.264)** |
| Red blood cell detection  Negative  Few  Moderate  **Many** | 100/213 (47%)  47/213 (22%)  16/213 (8%)  **50/213 (23%)** | 96/150 (64%)  32/150 (21%)  12/150 (8%)  **10/150 (7%)** | 1  1.410 (0.830, 2.394)  1.280 (0.576, 2.846)  **4.800 (2.303, 10.005)** | 1  1.515 (0.881, 2.606)  1.188 (0.527, 2.676)  **5.061 (2.379, 10.770)** |
| Prior medication used | 95 (42%) | 84 (52%) | 0.682 (0.455, 1.023) | 0.665 (0.442, 1.003) |
| Prior antibiotic used | 75 (33%) | 67 (41%) | 0.712 (0.469, 1.080) | 0.683 (0.448, 1.042) |

| *multivariate logistic regression including age, sex and categorical variable for length of time in Thailand  **multivariate logistic regression including age and categorical variable for length of time in Thailand  ^†^multivariate logistic regression including sex and categorical variable for length of time in Thailand  ^††^multivariate logistic regression including age and sex |
| --- |

Table S2. Crude and adjusted odds ratios for factors associated with bacterial pathogen detection in travelers’ diarrhea cases

|  | Case (n=389) | Control (n=400) |  | |
| --- | --- | --- | --- | --- |
|  | Number (%) | Number (%) | Crude OR (95% CI) | Adjusted OR* (95% CI) |
| **Bacterial pathogen detected** | **198 (51%)** | **118 (30%)** | **2.477 (1.849, 3.320)** | **2.248 (1.628, 3.103)** |

|  | Case with Bacterial Pathogen Detection (n=198) | Case without Bacterial Pathogen Detection (n=191) |  | |
| --- | --- | --- | --- | --- |
|  | Number (%) | Number (%) | Crude OR (95% CI) | Adjusted OR (95% CI)* |
| Sex (female) | 78 (39%) | 69 (36%) | 1.149 (0.762, 1.732) | 1.063 (0.698, 1.619)** |
| Traveler ≥ 25 years old | 154 (78%) | 168 (88%) | 0.479 (0.277, 0.830) | 0.512 (0.291, 0.904)^†^ |
| Nationality  Asian  European/North American  Oceania | 110 (56%)  78 (39%)  10 (5%) | 88 (46%)  84 (44%)  19 (10%) | 1  0.743 (0.490, 1.127)  0.421 (0.186, 0.952) | 1  0.810 (0.526, 1.246)  0.417 (0.183, 0.953) |
| Time in Thailand  0 - 29 days  30 - 89 days  90 - 364 days  365 - 729 days  730+ days | 113 (57%)  13 (7%)  22 (11%)  12 (6%)  38 (19%) | 94 (49%)  12 (6%)  27 (14%)  13 (7%)  45 (24%) | 1  0.901 (0.393, 2.069)  0.678 (0.362, 1.267)  0.768 (0.335, 1.763)  0.702 (0.421, 1.171) | 1  0.872 (0.379, 2.008)^††^  0.684 (0.364, 1.286)^††^  0.753 (0.328, 1.733)^††^  0.743 (0.443, 1.247)^††^ |
| Season  Hot  Rainy  Cool | 87 (44%)  85 (43%)  26 (13%) | 84 (44%)  70 (37%)  37 (19%) | 1  1.172 (0.758, 1.813)  0.678 (0.378, 1.217) | 1  1.169 (0.753, 1.815)  0.711 (0.394, 1.283) |
| Diarrhea duration  ≤ 1 day  2 days  3 days | 98 (49%)  43 (22%)  57 (29%) | 87 (46%)  33 (17%)  71 (37%) | 1  1.157 (0.676, 1.980)  0.713 (0.453, 1.121) | 1  1.169 (0.671, 2.035)  0.722 (0.455, 1.144) |
| Bowel movements/day  (mean (95%CI)) | 8.535 (7.940, 9.131) | 8.147 (7.590, 8.703) | 1.024 (0.975, 1.076) | 1.024 (0.974, 1.078) |
| Stool Characteristics  Watery  Loose  Mucous  Bloody | 147 (74%)  51 (26%)  39 (20%)  10 (5%) | 147 (77%)  44 (23%)  41 (21%)  6 (3%) | 0.863 (0.543, 1.372)  1.159 (0.729, 1.843)  0.897 (0.549, 1.468)  1.640 (0.584, 4.604) | 0.875 (0.547, 1.400)  1.143 (0.714, 1.828)  0.906 (0.551, 1.489)  1.711 (0.599, 4.889) |
| Symptoms  Fever  **Abdominal pain**  Nausea  Vomiting  Fatigue | 114 (58%)  **175 (88%)**  140 (71%)  108 (55%)  102 (52%) | 112 (59%)  **152 (80%)**  130 (68%)  99 (52%)  94 (49%) | 0.957 (0.640, 1.432)  **1.952 (1.116, 3.415)**  1.133 (0.736, 1.744)  1.115 (0.749, 1.661)  1.096 (0.737, 1.632) | 0.957 (0.633, 1.448)  **1.928 (1.096, 3.392)**  1.072 (0.688, 1.668)  1.068 (0.710, 1.606)  1.093 (0.731, 1.634) |
| White blood cell detection  Negative  Few  Moderate  **Many** | 52/185 (28%)  39/185 (21%)  26/185 (14%)  **68/185 (37%)** | 80/178 (45%)  45/178 (25%)  23/178 (13%)  **30/178 (17%)** | 1  1.333 (0.767, 2.318)  1.739 (0.898, 3.368)  **3.487 (2.005, 6.066)** | 1  1.271 (0.725, 2.229)  1.568 (0.794, 3.094)  **3.477 (1.978, 6.114)** |
| Red blood cell detection  Negative  Few  Moderate  **Many** | 84/185 (45%)  42/185 (23%)  13/185 (7%)  **46/185 (25%)** | 112/178 (63%)  37/178 (21%)  15/178 (8%)  **14/178 (8%)** | 1  1.514 (0.896, 2.558)  1.156 (0.522, 2.558)  **4.381 (2.260, 8.491)** | 1  1.601 (0.935, 2.744)  1.041 (0.463, 2.340)  **4.723 (2.371, 9.408)** |
| Prior medication used | 83 (42%) | 96 (50%) | 0.714 (0.479, 1.066) | 0.687 (0.458, 1.031) |
| Prior antibiotic used | 65 (33%) | 77 (40%) | 0.724 (0.478, 1.095) | 0.696 (0.457, 1.059) |

| *multivariate logistic regression including age, sex and categorical variable for length of time in Thailand  **multivariate logistic regression including age and categorical variable for length of time in Thailand  ^†^multivariate logistic regression including sex and categorical variable for length of time in Thailand  ^††^multivariate logistic regression including age and sex |
| --- |

Table S3. Crude and adjusted odds ratios for factors associated with norovirus detection in travelers’ diarrhea cases

|  | Case (n=259) | Control (n=292) |  | |
| --- | --- | --- | --- | --- |
|  | Number (%) | Number (%) | Crude OR (95% CI) | Adjusted OR (95% CI)* |
| **Norovirus detected** | **32 (12%)** | **1 (0.3%)** | **41.022 (5.563, 302.480)** | **35.903 (4.740, 271.947)** |

|  | Case with norovirus detection (n=32) | Case without norovirus detection (n=227) |  | |
| --- | --- | --- | --- | --- |
|  | Number (%) | Number (%) | Crude OR (95% CI) | Adjusted OR (95% CI)* |
| Sex (female) | 14 (44%) | 80 (35%) | 1.429 (0.675, 3.024) | 1.426 (0.663, 3.068)** |
| Traveler ≥ 25 years old) | 25 (78%) | 194 (85%) | 0.608 (0.243, 1.518) | 0.587 (0.226, 1.523)^†^ |
| Nationality  Asian  European/North American  Oceania | 15 (47%)  13 (41%)  4 (13%) | 102 (45%)  110 (48%)  15 (7%) | 1  0.804 (0.365, 1.771)  1.813 (0.531, 6.197) | 1  0.854 (0.380, 1.919)  1.965 (0.554, 6.974) |
| Time in Thailand  0 - 29 days  30 - 89 days  90 - 364 days  365 - 729 days  730+ days | 15 (47%)  3 (9%)  3 (9%)  1 (3%)  10 (31%) | 113 (50%)  15 (7%)  25 (11%)  16 (7%)  58 (26%) | 1  1.507 (0.390, 5.821)  0.904 (0.243, 3.361)  0.471 (0.058, 3.810)  1.299 (0.549, 3.071) | 1  1.403 (0.359, 5.482)^††^  0.939 (0.249, 3.546)^††^  0.441 (0.054, 3.588)^††^  1.403 (0.585, 3.365)^††^ |
| Season  Hot  Rainy  Cool | 13 (41%)  15 (47%)  4 (13%) | 97 (43%)  90 (40%)  40 (18%) | 1  1.244 (0.561, 2.757)  0.746 (0.229, 2.427) | 1  1.171 (0.520, 2.634)  0.722 (0.220, 2.371) |
| Diarrhea duration  ≤ 1 day  2 days  **3 days** | 21 (66%)  5 (16%)  **6 (19%)** | 91 (40%)  48 (21%)  **88 (39%)** | 1  0.451 (0.160, 1.272)  **0.295 (0.114, 0.767)** | 1  0.433 (0.149, 1.260)  **0.266 (0.099, 0.710)** |
| Bowel movements/day  (mean (95%CI)) | 8.219 (7.069, 9.369) | 8.317 (7.778, 8.857) | 0.994 (0.905, 1.092) | 1.010 (0.918, 1.111) |
| Stool characteristics  Watery  Loose  Mucous  Bloody | 26 (81%)  6 (19%)  8 (25%)  0 (0%) | 170 (75%)  57 (25%)  47 (21%)  9 (4%) | 1.453 (0.569, 3.708)  0.688 (0.270, 1.757)  1.277 (0.539, 3.023)  - | 1.567 (0.605, 4.063)  0.638 (0.246, 1.654)  1.274 (0.532, 3.051)  - |
| Symptoms  Fever  Abdominal pain  Nausea  **Vomiting**  Fatigue | 23 (72%)  26 (81%)  26 (81%)  **25 (78%)**  21 (66%) | 143 (63%)  190 (84%)  153 (67%)  **107 (47%)**  118 (52%) | 1.501 (0.664, 3.396)  0.844 (0.325, 2.193)  2.096 (0.827, 5.312)  **4.005 (1.665, 9.634)**  1.763 (0.813, 3.826) | 1.704 (0.735, 3.952)  0.825 (0.315, 2.160)  2.137 (0.829, 5.508)  **4.099 (1.688, 9.954)**  1.841 (0.835, 4.058) |
| White blood cell detection  Negative  Few  Moderate  Many | 10 (31%)  9 (28%)  6 (19%)  7 (22%) | 86 (38%)  48 (21%)  24 (11%)  69 (30%) | 1  1.613 (0.613, 4.242)  2.150 (0.710, 6.515)  0.872 (0.316, 2.411) | 1  1.749 (0.651, 4.697)  2.294 (0.723, 7.278)  0.925 (0.327, 2.621) |
| Red blood cell detection  Negative  Few  Moderate  Many | 18 (56%)  5 (16%)  5 (16%)  4 (13%) | 120 (53%)  50 (22%)  12 (5%)  45 (20%) | 1  0.667 (0.235, 1.894)  2.778 (0.875, 8.816)  0.593 (0.190, 1.846) | 1  0.725 (0.251, 2.093)  3.145 (0.946, 10.455)  0.575 (0.175, 1.887) |
| Prior medication used | 17 (53%) | 95 (42%) | 1.575 (0.749, 3.309) | 1.606 (0.759, 3.399) |
| Prior antibiotic used | 13 (41%) | 72 (32%) | 1.473 (0.690, 3.146) | 1.400 (0.651, 3.014) |

| *multivariate logistic regression including age, sex and categorical variable for length of time in Thailand  **multivariate logistic regression including age and categorical variable for length of time in Thailand  ^†^multivariate logistic regression including sex and categorical variable for length of time in Thailand  ^††^multivariate logistic regression including age and sex |
| --- |

Table S4. Crude and adjusted odds ratios for factors associated with protozoa detection in travelers’ diarrhea cases

|  | Case (n=389) | Control (n=400) |  | |
| --- | --- | --- | --- | --- |
|  | Number (%) | Number (%) | Crude OR (95% CI) | Adjusted OR (95% CI)* |
| Protozoa detected | 13 (3%) | 8 (2%) | 1.694 (0.694, 4.134) | 1.658 (0.610, 4.505) |

|  | Case with protozoa detection (n=13) | Case without protozoa detection (n=376) |  | |
| --- | --- | --- | --- | --- |
|  | Number (%) | Number (%) | Crude OR (95% CI) | Adjusted OR (95% CI)* |
| Sex (female) | 3 (23%) | 144 (38%) | 0.483 (0.131, 1.786) | 0.430 (0.113, 1.640)** |
| Traveler ≥ 25 years old | 10 (77%) | 312 (83%) | 0.684 (0.183, 2.554) | 0.608 (0.152, 2.426)^†^ |
| Nationality  Asian  European/North American  Oceania | 7 (54%)  6 (46%)  0 (0%) | 191 (51%)  156 (41%)  29 (8%) | 1  1.049 (0.346, 3.187)  - | 1  1.279 (0.404, 4.050)  - |
| Time in Thailand  0 - 29 days  30 - 89 days  90 - 364 days  365 - 729 days  730+ days | 8 (62%)  0 (0%)  2 (15%)  0 (0%)  3 (23%) | 199 (53%)  25 (7%)  47 (13%)  25 (7%)  80 (21%) | 1  -  1.059 (0.218, 5.148)  -  0.933 (0.241, 3.606) | 1  -  0.952 (0.192, 4.730)^††^  -  1.103 (0.272, 4.478)^††^ |
| Season  Hot  **Rainy**  **Cool** | 1 (8%)  **8 (62%)**  **4 (31%)** | 170 (45%)  **1 (0.3%)**  **59 (16%)** | 1  **9.252 (1.144, 74.840)**  **11.525 (1.263, 105.192)** | 1  **10.059 (1.226, 82.519)**  **14.199 (1.523, 132.378)** |
| Diarrhea duration  ≤ 1 day  2 days  **3 days** | 3 (23%)  1 (8%)  **9 (69%)** | 182 (48%)  75 (20%)  **119 (32%)** | 1  0.809 (0.083, 7.901)  **4.588 (1.217, 17.295)** | 1  0.745 (0.074, 7.483)  **5.003 (1.302, 19.223)** |
| Bowel movements/day  (mean (95%CI)) | 7.846 (5.860, 9.833) | 8.362 (7.945, 8.778) | 0.966 (0.829, 1.125) | 0.972 (0.839, 1.127) |
| Stool characteristics  Watery  Loose  Mucous  Bloody | 10 (77%)  3 (23%)  3 (23%)  0 (0%) | 284 (76%)  92 (24%)  77 (20%)  16 (4%) | 1.080 (0.291, 4.008)  0.926 (0.250, 3.437)  1.165 (0.313, 4.336)  - | 1.045 (0.274, 3.977)  0.957 (0.251, 3.646)  1.352 (0.354, 5.159)  - |
| Symptoms  Fever  **Abdominal pain**  Nausea  Vomiting  Fatigue | 7 (54%)  **6 (46%)**  6 (46%)  6 (46%)  6 (46%) | 219 (58%)  **321 (85%)**  264 (70%)  201 (53%)  190 (51%) | 0.836 (0.276, 2.537)  **0.147 (0.048, 0.453)**  0.364 (0.120, 1.106)  0.746 (0.246, 2.262)  0.839 (0.277, 2.544) | 0.806 (0.260, 2.504)  **0.139 (0.044, 0.441)**  0.350 (0.111, 1.109)  0.782 (0.248, 2.463)  0.740 (0.239, 2.297) |
| White blood cell detection  Negative  Few  Moderate  Many | 3 (23%)  5 (38%)  4 (31%)  1 (8%) | 129/350 (37%)  79/350 (23%)  45/350 (13%)  97/350 (28%) | 1  2.722 (0.633, 11.701)  3.822 (0.824, 17.739)  0.443 (0.045, 4.327) | 1  2.412 (0.547, 10.634)  3.779 (0.766, 18.647)  0.506 (0.051, 5.008) |
| Red blood cell detection  Negative  Few  Moderate  Many | 10 (77%)  3 (23%)  0 (0%)  0 (0%) | 186/350 (53%)  76/350 (22%)  28/350 (8%)  60/350 (17%) | 1  0.734 (0.197, 2.742)  -  - | 1  0.721 (0.189, 2.755)  -  - |
| Prior medication used | 7 (54%) | 172 (46%) | 1.384 (0.456, 4.195) | 1.268 (0.411, 3.911) |
| Prior antibiotic used | 5 (38%) | 137 (36%) | 1.090 (0.350, 3.399) | 1.040 (0.328, 3.294) |
| Risk country destination  Low risk-Bangkok  Moderate  **High risk** | 8 (62%)  2 (15%)  **3 (23%)** | 252 (67%)  116 (31%)  **8 (2%)** | 1  0.543 (0.114, 2.598)  **11.813 (2.630, 53.048)** | 1  0.545 (0.110, 2.698)  **11.061 (2.100, 58.241)** |

| *multivariate logistic regression including age, sex and categorical variable for length of time in Thailand  **multivariate logistic regression including age and categorical variable for length of time in Thailand  ^†^multivariate logistic regression including sex and categorical variable for length of time in Thailand  ^††^multivariate logistic regression including age and sex |
| --- |

Table S5. Crude and adjusted odds ratios for bacterial pathogens and symptoms associated with region of origin in travelers’ diarrhea cases

|  | Asian Cases (n=198) | Non-Asian Cases (n=191) |  |  |
| --- | --- | --- | --- | --- |
|  | Number (%) | Number (%) | Crude OR (95% CI) | Adjusted OR (95% CI)* |
| Bacteria  Campylobacter  Salmonella  Shigella  Aeromonas  Plesiomonas  **Vibrio**  ETEC | 20 (10%)  26 (13%)  5 (3%)  9 (5%)  33 (17%)  **39 (20%)**  18/173 (10%) | 33 (17%)  20 (10%)  7 (4%)  9 (5%)  22 (12%)  **14 (7%)**  8/152 (5%) | 0.538 (0.297, 0.976)  1.292 (0.695, 2.403)  0.681 (0.212, 2.184)  0.963 (0.374, 2.480)  1.536 (0.860, 2.745)  **3.101 (1.624, 5.923)**  2.090 (0.882, 4.955) | 0.518 (0.277, 0.969)  1.166 (0.615, 2.212)  0.488 (0.146, 1.632)  0.907 (0.343, 2.404)  1.395 (0.767, 2.537)  **3.372 (1.716, 6.627)**  1.747 (0.718, 4.252) |
| Symptoms  Fever  Nausea  Vomiting  Abdominal pain  Fatigue | 102 (52%)  129 (65%)  99 (50%)  170 (86%)  85 (43%) | 124 (65%)  141 (74%)  108 (57%)  157 (82%)  111 (58%) | 0.574 (0.382, 0.863)  0.663 (0.429, 1.025)  0.769 (0.516, 1.146)  1.315 (0.762, 2.268)  0.542 (0.363, 0.811) | 0.574 (0.374, 0.879)  0.556 (0.350, 0.884)  0.700 (0.460, 1.066)  1.315 (0.751, 2.303)  0.532 (0.355, 0.806) |

|  | North American/European Cases (n=162) | Non-North American/European Cases (n=227) |  |  |
| --- | --- | --- | --- | --- |
|  | Number (%) | Number (%) | Crude OR (95% CI) | Adjusted OR (95% CI)* |
| Bacteria  **Campylobacter**  Salmonella  Shigella  Aeromonas  Plesiomonas  Vibrio  ETEC | **33 (20%)**  12 (7%)  7 (4%)  7 (4%)  20 (12%)  13 (8%)  7/131 (5%) | **20 (9%)**  34 (15%)  5 (2%)  11 (5%)  35 (15%)  40 (18%)  19/194 (10%) | **2.648 (1.457, 4.812)**  0.454 (0.227, 0.907)  2.005 (0.625, 6.434)  0.887 (0.336, 2.339)  0.773 (0.428, 1.395)  0.408 (0.210, 0.791)  0.520 (0.212, 1.275) | **2.792 (1.489, 5.236)**  0.506 (0.249, 1.030)  2.768 (0.821, 9.332)  0.915 (0.336, 2.491)  0.864 (0.469, 1.591)  0.395 (0.198, 0.786)  0.628 (0.247, 1.594) |
| Symptoms  Fever  Nausea  Vomiting  Abdominal pain  Fatigue | 101 (62%)  114 (70%)  86 (53%)  131 (81%)  95 (59%) | 125 (55%)  156 (69%)  121 (53%)  196 (86%)  101 (44%) | 1.351 (0.895, 2.039)  1.081 (0.697, 1.676)  0.991 (0.662, 1.485)  0.668 (0.388, 1.152)  1.769 (1.177, 2.659) | 1.302 (0.845, 2.006)  1.284 (0.809, 2.038)  1.100 (0.719, 1.681)  0.662 (0.377, 1.162)  1.803 (1.181, 2.751) |

|  | Australian/New Zealander Cases (n=29) | Non-Australian/New Zealander Cases (n=360) |  |  |
| --- | --- | --- | --- | --- |
|  | Number (%) | Number (%) | Crude OR (95% CI) | Adjusted OR (95% CI)* |
| Bacteria  Campylobacter  **Salmonella**  Shigella  Aeromonas  Plesiomonas  Vibrio  ETEC | 0 (0%)  **8 (28%)**  0 (0%)  2 (7%)  2 (7%)  1 (3%)  1/21 (5%) | 53 (15%)  **38 (11%)**  12 (3%)  16 (4%)  53 (15%)  52 (14%)  25/304 (8%) | - **  **3.228 (1.338, 7.790)**  -  1.593 (0.348, 7.291)  0.429 (0.099, 1.858)  0.212 (0.028, 1.589)  0.558 (0.072, 4.333) | -  **3.016 (1.222, 7.442)**  -  1.737 (0.367, 8.225)  0.421 (0.096, 1.844)  0.184 (0.024, 1.395)  0.548 (0.069, 4.355) |
| Symptoms  **Fever**  **Nausea**  **Vomiting**  Abdominal pain  Fatigue | **23 (79%)**  **27 (93%)**  **22 (76%)**  26 (90%)  16 (55%) | **203 (56%)**  **243 (68%)**  **185 (51%)**  301 (84%)  180 (50%) | **2.965 (1.179, 7.456)**  **6.500 (1.520, 27.798)**  **2.973 (1.239, 7.134)**  1.699 (0.498, 5.796)  1.231 (0.575, 2.633) | **3.252 (1.270, 8.325)**  **6.358 (1.459, 27.713)**  **2.790 (1.139, 6.834)**  1.710 (0.496, 5.896)  1.237 (0.572, 2.673) |
| *multivariate logistic regression including age, sex and categorical variable for length of time in Thailand  ** p=0.021, Fisher’s Exact Test, 2-sided | | | | |
